# Association of IFIH1 and pro-inflammatory mediators: Potential new clues in SLE-associated pathogenesis

**DOI:** 10.1371/journal.pone.0171193

**Published:** 2017-02-24

**Authors:** Melissa E. Munroe, Nathan Pezant, Michael A. Brown, Dustin A. Fife, Joel M. Guthridge, Jennifer A. Kelly, Graham Wiley, Patrick M. Gaffney, Judith A. James, Courtney G. Montgomery

**Affiliations:** 1 Arthritis and Clinical Immunology Research Program, Oklahoma Medical Research Foundation, Oklahoma City, OK, United States of America; 2 Department of Biostatistics and Epidemiology, University of Oklahoma Health Sciences Center, Oklahoma City, OK, United States of America; 3 Department of Medicine and Pathology, University of Oklahoma Health Sciences Center, Oklahoma City, OK, United States of America; Instituto Nacional de Ciencias Medicas y Nutricion Salvador Zubiran, MEXICO

## Abstract

Antiviral defenses are inappropriately activated in systemic lupus erythematosus (SLE) and association between SLE and the antiviral helicase gene, *IFIH1*, is well established. We sought to extend the previously reported association of pathogenic soluble mediators and autoantibodies with mouse *Mda5* to its human ortholog, *IFIH1*. To better understand the role this gene plays in human lupus, we assessed association of *IFIH1* variants with soluble mediators and autoantibodies in 357 European-American SLE patients, first-degree relatives, and unrelated, unaffected healthy controls. Association between each of 135 genotyped SNPs in *IFIH1* and four lupus-associated plasma mediators, IL-6, TNF-α, IFN-β, and IP-10, were investigated via linear regression. No significant associations were found to SNPs orthologous to those identified in exon 13 of the mouse. However, outside of this region there were significant associations between IL-6 and rs76162067 (*p = 0*.*008*), as well as IP-10 and rs79711023 (*p = 0*.*003*), located in a region of *IFIH1* previously shown to directly influence MDA-5 mediated IP-10 and IL-6 secretion. SLE patients and FDRs carrying the minor allele for rs79711023 demonstrated lower levels of IP-10, while only FDRs carrying the minor allele for rs76162067 demonstrated an increased level of IL-6. This would suggest that the change in IP-10 is genotypically driven, while the change in IL-6 may be reflective of SLE transition status. These data suggest that *IFIH1* may contribute to SLE pathogenesis via altered inflammatory mechanisms.

## Introduction

Systemic lupus erythematosus (SLE) is an autoimmune disease marked by immune dysregulation and chronic inflammation resulting from reduced immunologic tolerance to nuclear self-antigens. One mechanism by which autoimmunity may develop is through an innate immune response to viral infection that results in cross-reactive recognition of self-antigen via molecular mimicry [1]. One component of antiviral immune defense is the family of retinoic acid-inducible gene-I-like receptors (RIG-I like receptors [RLRs]). RLRs are encoded by a number of IFN-inducible genes [2]. One such IFN-inducible gene, Interferon induced with helicase C domain 1 (*IFIH1*), has previously been associated with multiple autoimmune conditions, including SLE [3, 4]. *IFIH1* has been associated in SLE with increased sensitivity to serum Type I IFN and the presence of anti-dsDNA autoantibodies [5].

In a recent study, melanoma-differentiation-associated gene 5 (*Mda5*), the mouse ortholog to *IFIH1*, was shown to be associated with IFN-β, IL-6, CXCL10 (IP-10), ISg56, TNF-α, anti-nuclear antibodies (ANA), and anti-dsDNA antibodies [2]. The *Mda5* mutant mice carrying a single missense mutation within exon 13 had significantly higher mRNA expression levels of IFN-β, IL-6, IP-10, ISg56, and TNF-α within their kidneys relative to congenic wild-type (WT) mice. There were also significant increases in serum ANA and anti-dsDNA antibodies in *Mda5* mutant mice compared to WT mice. Coinciding with an increase in these pro-inflammatory mediators, *Mda5* mutant mice spontaneously developed lupus-like nephritis and systemic autoimmune symptoms without viral infection.

The findings of Funabiki et al. link *Mda5* with dysregulated immune function resulting in lupus-like disease [2]. As these associations have yet to be investigated in human SLE, we sought to determine if *IFIH1*, the human ortholog of *Mda5*, is associated with altered soluble mediators and autoantibodies in SLE patients compared to unaffected first-degree relatives of SLE patients (FDRs) and unrelated, unaffected controls with no family history of SLE. We have recently demonstrated immunologic differences between FDRs who remained unaffected during a follow-up period of over six years vs. those who have transitioned to classified SLE and unrelated, unaffected healthy individuals [6].

## Materials and methods

### Study population

Experiments were performed in accordance with the Helsinki Declaration and approved by the Institutional Review Board (IRB) of the Oklahoma Medical Research Foundation (OMRF). Appropriately consented, retrospectively collected, and de-identified clinical and genotyping data, as well as serum and plasma samples, were utilized for the study; the study was approved by the OMRF IRB under exemption 45 CFR 46.101(b) (4). A total of 357 EA study participants, including SLE patients meeting >4 cumulative ACR criteria [7], unaffected first degree relatives of SLE patients (FDRs) not included in the current study, and unrelated, unaffected controls with no family history of SLE, were retrospectively selected from the Oklahoma Rheumatic Disease Research Core Center (ORDRCC) (**Table 1**). Individuals donating samples to the ORDRCC completed IRB-approved, written consent forms prior to sample procurement. Written consent was recorded into the electronic, de-identified ORDRCC database; a copy of the signed consent form was given to each ORDRCC participant and the original document retained by the clinical coordinator. These individuals were selected based on the availability of retrospectively collected, appropriately consented, genotyping data, as well as serum and plasma samples, to assess levels of soluble mediators and the presence of lupus-associated autoantibody specificities. The inclusion of FDRs in addition to cases and controls boosted the power of our analysis from 0.25 (cases only) to 0.80 (cases + FDRs + controls), f^2^ effect size = 0.05. For the assessment of association between genetic variants and a quantitative trait (as presented here), the classification of an individual as diseased or not is only relevant in that it 1) helps to identify individuals that may be on treatment affecting levels of the quantitative trait being examined and 2) it allows for assurance that the distribution of the trait being examined is as inclusive as possible. Thus, for our purposes, FDRs, which are known to have elevated levels of many autoantibodies and soluble mediators [6, 8, 9], were included to boost the power, represent a larger range of the trait distribution and allow us to assess if treatment was influencing the values of the cytokines or autoantibodies being studied.

**Table 1:** European-American Study Participants.

Disease Status	SLE^b^	FDR^c^	Ctl^d^
**n (%**^**a**^**)**	128 (36)	144 (40)	85 (24)
**Gender: n (%)**			
• Female	121 (95)	130 (90)	82 (96)
• Male	7 (5)	14 (10)	3 (4)
**Age: Median (Interquartile Range)**	48 (34–54)	49 (40–58)	53 (52–61)

^a^ Percent of 357 total participants.

^b^ SLE = patients meeting ≥ 4 ACR criteria.

^c^ FDR = first degree relatives of SLE patients meeting < 4 ACR criteria.

^d^ Ctl = unrelated, unaffected controls with no family history of SLE.

### Genotyping

Samples were genotyped on the Illumina ImmunoChip array on an Illumina iScan according to the manufacturers protocols [10]. Genotypes were called using Opticall [11] using the default options with the addition of -nointcutoff option in order to manually remove intensity outliers. Genotype clusters were determined using Evoker [12]. Standard quality control procedures were as follows: removal of samples with mean intensity values <0.25 or >3; removal of SNPs and samples with call rates <90%; removal of samples with heterozygosity >3 sigma; and removal of related samples. Principal component analysis was performed using EIGENSTRAT and genetic outliers were removed as described in [13]. Insertion/deletions were also removed prior to analyzing the data. After quality control, 135 observed SNPs remained and no imputation was performed.

### Detection of plasma soluble mediators

Plasma levels of IL-6, TNF-α, IFN-β, and IP-10 (CXCL10) were assessed by xMAP multiplex assay (eBioscience/Affymetrix, Santa Clara, CA), utilizing a single lot of assay plates to limit lot-specific assay variability [14]. In addition, SLE patient, FDR, and control samples were included on the same assay plate; a known control serum was included on each plate (Cellgro human AB serum, Cat#2931949, L/N#M1016) to limit inter-plate variability. Data were obtained using the Bio-Rad BioPlex 200^®^ array system (Bio-Rad Technologies, Hercules, CA), with a lower boundary of 100 beads per analyte per sample. Analyte concentrations were interpolated from 5-parameter logistic nonlinear regression standard curves. Analytes below the detection limit were assigned a value of 0.001 pg/ml. Well-specific validity was assessed by AssayCheX™ QC microspheres (Radix BioSolutions, Georgetown, TX, USA) to evaluate non-specific binding. Mean inter-assay coefficient of variance (CV) of multiplexed bead-based assays for cytokine detection has previously been shown to be 10–14% [15, 16], and a similar average CV (11%) was obtained across the analytes in this assay using healthy control serum. Intra-assay precision of duplicate wells averaged <10% CV in each multiplex assay.

### Detection of SLE-associated autoantibodies

Serum and plasma samples were screened for autoantibody specificities using the BioPlex 2200 multiplex system (Bio-Rad Technologies). The BioPlex 2200 ANA kit uses fluorescently dyed magnetic beads for simultaneous detection of 11 autoantibody specificities, including SLE-associated autoantibodies against dsDNA, chromatin, Ro/SSA, La/SSB, Sm, the Sm/RNP complex, and RNP[9]. Anti-dsDNA (IU/mL) has a previously determined positive cutoff of 10 IU/mL; an Antibody Index (AI) value (range 0–8) is reported by the manufacturer to reflect the fluorescence intensity of each of the other autoantibody specificities with a positive cutoff as AI = 1.0. The AI scale is standardized relative to calibrators and control samples provided by the manufacturer.

### Statistical methods

Power calculations were completed using the powr.f2.test() function in the “pwr” package in R (version 1.1–3) [17]. *IFIH1* association analyses of soluble mediators (IL-6, TNF-α, IFN-β, and IP-10) and lupus-associated autoantibody (anti-dsDNA and ANA [positive for any of the following autoantibody specificities: dsDNA, chromatin, Ro/SSA, La/SSB, Sm, SmRNP, nRNP) data obtained via xMAP multiplex assays were restricted to those previously investigated by Funabiki et al. [2]. Soluble mediator and autoantibody data were transformed via Box-Cox transformation and linear regression analysis was used to assess associations between transformed values and each individual SNP. Age and gender were included as covariates and an additive model of inheritance was assumed in the linear regression analyses.

Association analysis of soluble mediator and autoantibody levels, as well as the number of detected SLE-associated autoantibody specificities, with SNPs in exon 13 of *IFIH1* was subsequently expanded to include the 135 quality controlled SNPs within the *IFIH1* gene. Linkage disequilibrium between novel significant SNPs and previously identified SLE-associated SNPs was analyzed using the LDmatrix module in LDlink with all European populations [18]. Differences in levels of soluble mediators between major and minor alleles were determined by unpaired t-test (transformed data) and Mann-Whitney test (untransformed concentration data). Differences in levels of soluble mediators between SLE patients (cases), unaffected FDRs, and unrelated, unaffected controls were determined by ANOVA with Tukey’s multiple comparison (transformed data) and Kruskal-Wallis with Dunn’s multiple comparison (untransformed concentration data). Differences in levels of soluble mediators between SLE patients (cases), unaffected FDRs, and unrelated, unaffected controls carrying either major (CC) or minor (TC) alleles of rs7316162067 or rs79711023 for IL-6 or IP-10 levels, respectively, were determined by Kruskal-Wallis with Dunn’s multiple comparison (untransformed concentration data).

## Results

**Fig 1:**
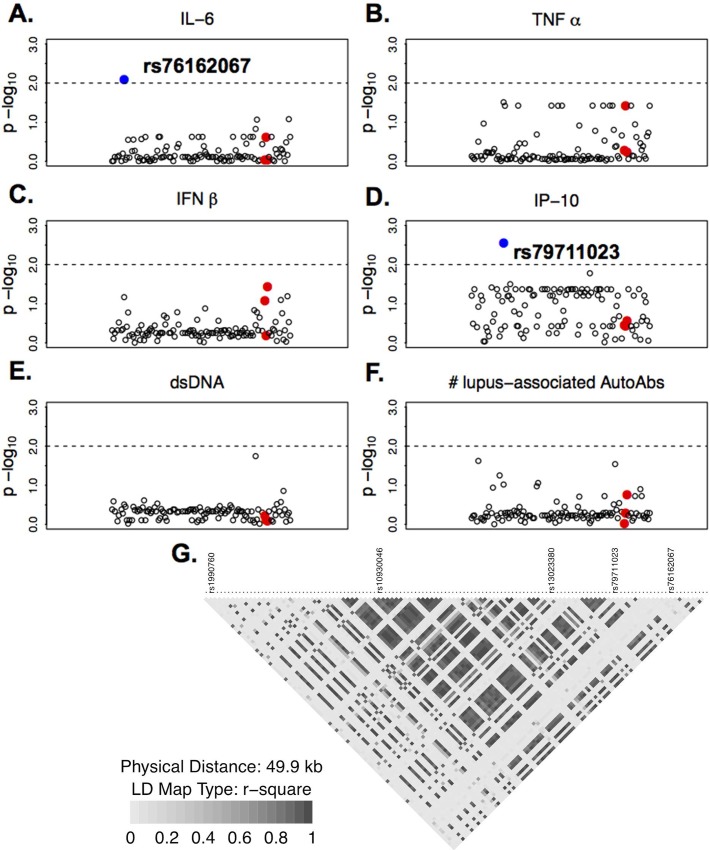
Genotypic Associations between *IFIH1* and Immune Mediators. Zoom plots of associations between SNPs in *IFIH1* and box-cox transformed plasma levels of (**A**) IL-6, (**B**) TNF-α, (**C**) IFN-β, (**D**) IP-10, (**E**) anti-dsDNA, and (**F**) total number of positive lupus-associated autoantibody specificities (dsDNA, chromatin, Ro/SSA, La/SSB, Sm, SmRNP, and nRNP, as described in *Materials and Methods*), displayed as negative log transformed p-values (y-axis). Red dots indicate SNPs within Exon 13. LD map of *IFIH1* SNPs is presented (**G**).

**Fig 2:**
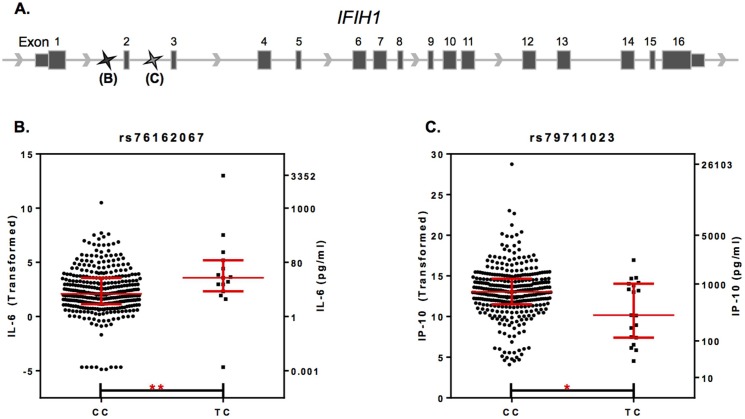
IFIH1 effect on plasma levels of IL-6 and IP-10. Exon map of IFIH1 is presented (**A**) containing labeled SNPs rs76162067 and rs79711023. IL-6 (**B**) and IP-10 (**C**) levels in study participants expressing the major (CC) or minor (CT) allele of SNP rs76162067 or rs79711023, respectively, are expressed as box-cox transformed (left y-axis) and concentration (right y-axis) values. Exon order is displayed L-R for ease of interpretation, although IFIH1 is transcribed R-L from the negative strand. Data in **B-C** are presented as median ± interquartile range. *p≤0.*05*, **p<0.*01* Mann-Whitney test.

We sought to determine if the soluble mediators that significantly associated with mutant *Mda5* [2], also associated with the human ortholog, *IFIH1*. Plasma levels of select soluble mediators and the presence of lupus-associated autoantibody specificities in 357 individuals (SLE patients, FDRs of SLE patients not included in this study, and unrelated, unaffected controls with no family history of SLE were analyzed along with 135 quality controlled SNPs in the region of *IFIH1* (**Fig 1**). The zoom plots presented demonstrate no significant associations (*p<0*.*01*) between variants within *IFIH1* exon 13 (red dots) and plasma levels of IL-6, TNF-α, IFN-β, IP-10, or anti-dsDNA (**Fig 1A–1E**). The number of positive lupus-associated autoantibody specificities was also not significantly associated with variants in exon 13 (**Fig 1F**). However, significant associations were found with SNPs outside of exon 13 (blue dots). IL-6 was significantly associated with rs76162067 (*p = 0*.*008*; **Fig 1A**), while IP-10 was significantly associated with rs79711023 (*p = 0*.*003*; **Fig 1D**), both of which are upstream of previously identified SLE-associated SNPs, rs13023380, rs10930046, and rs1990760 ([4, 5], **Fig 1G**). Results from LDlink show that neither rs76162067 nor rs79711023 are in strong linkage disequilibrium with any of the previously identified SLE-associated SNPs mentioned above (**S1 Table**). Of the 357 individuals assessed, 14 carried the minor (TC) allele for rs73162067, an intronic SNP located between exons 1 and 2, while 19 individuals carried the minor (TC) allele for rs79711023, an intronic SNP located between exon 2 and 3 (**Fig 2A**). Those individuals carrying the minor allele for rs76162067 and rs79711023 exhibited higher plasma levels of IL-6 (*p = 0*.*0086*) and lower plasma levels of IP-10 (*p = 0*.*0250*), respectively (**Fig 2B and 2C**). These data confirm in humans the presence of significant associations between *IFIH1* and distinct pro-inflammatory mediators.

**Fig 3:**
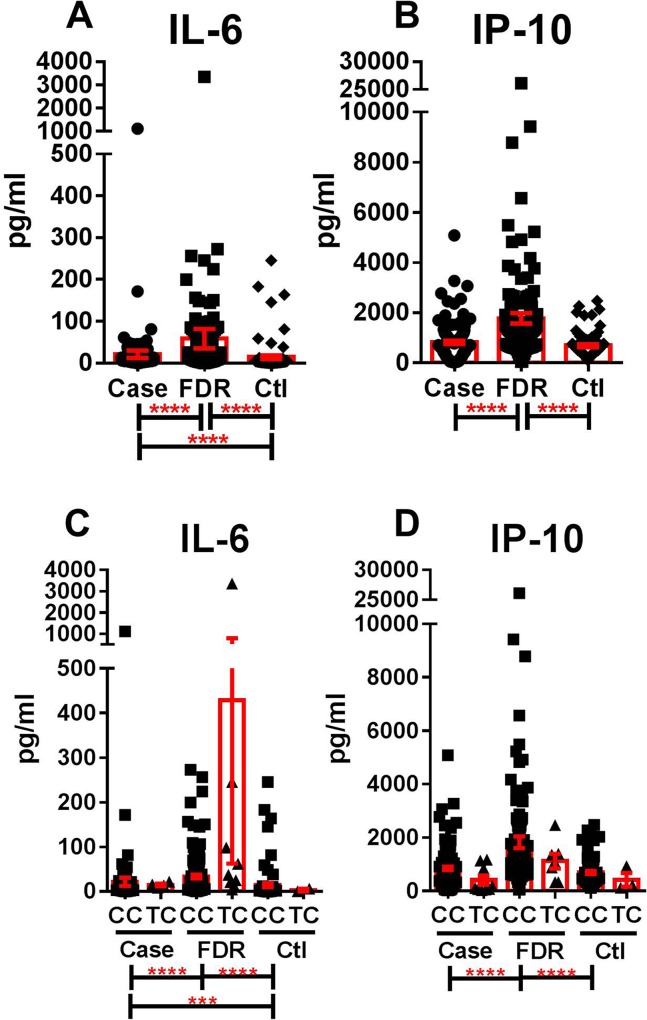
Altered IL-6 and IP-10 levels in SLE patients and lupus relatives. Plasma levels of IL-6 (**A, C**) and IP-10 (**B, D**) were assessed in SLE patients (case), unaffected first-degree relatives of SLE patients (FDR), and unrelated, unaffected controls with no family history of SLE (Ctl). IL-6 (**C**) and IP-10 (**D**) levels are compared by genotype of associated SNPs rs76162067 and rs79711023, respectively. Data are presented as mean + SEM. *p<0.*05*, **p<0.*01*, ***p<0.*001*, ****p<0.*0001* by Kruskal-Wallis test with Dunn’s multiple comparison for all groups assessed (Case/FDR/Ctl [**A-B**]; Case/FDR/Ctl carrying major (CC) or minor (TC) alleles [**C-D**]). Only comparisons found to be statistically significant were marked.

We next evaluated IL-6 and IP-10 levels by affectation status in SLE patients compared to unaffected FDRs and unrelated controls (**Fig 3**). FDRs had the highest plasma levels of both IL-6 (**Fig 3A**) and IP-10 (**Fig 3B**), significantly higher than both cases and unrelated controls (*p<0*.*0001)*. Cases had significantly higher plasma levels of IL-6 than unrelated controls (*p<0*.*0001*). This pattern was unchanged by the presence of major (CC) or minor (TC) alleles of rs7316162067 (**Fig 3C**) or rs79711023 (**Fig 3D**) for IL-6 or IP-10 levels, respectively. Although SLE patients were more likely than FDRs and Ctls to have a history of prednisone, hydroxychloroquine, or immunosuppressant medication usage (*p<0*.*0001*, **S2 Table**), neither the presence nor absence of these medications altered the *pattern* of IL-6 and IP-10 levels in FDRs, SLE patients, and controls, such that FDRs continued to have the highest levels of IL-6 and IP-10, followed by cases and controls (**S1 Fig**). Although immunosuppressent use decreased IL-6 and IP-10 levels, while prednisone use increased these cytokine levels in cases alone (**S1 Fig**), correcting for the presence of treatment did not alter the significance of the previous association analysis (rs76162067, *p = 0*.*016*; rs76162067, *p = 0*.*024*). Finally, other soluble mediators that were not found to be associated with SNPs evaluated within *IFIH1*, including TNF-α (**Fig 1B**) and IFN-β (**Fig 1C**), were also highest in FDRs, followed by SLE patients (cases) and controls (**S2A and S2B Fig**). In addition to neither anti-dsDNA levels (**Fig 1E**), nor number of SLE-associated autoantibody specificities (**Fig 1F**) associated with IFIH1 (**Fig 1E**), there were no difference in anti-dsDNA levels between SLE patients, FDRs, and controls (**S2C Fig**). However, cases did have significant accumulation of SLE-associated autoantibody specificities compared to FDRs (*p = 0*.*004*) and controls (*p = 0*.*012*) (**S2D Fig**). These data suggest that *IFIH1*-independent effects are present that contribute to altered pro-inflammatory mediator levels and the presence of SLE-associated autoantibodies.

## Discussion

We sought to determine if pro-inflammatory mediators previously shown to be associated with *Mda5*, resulting in a lupus-like phenotype in mice [2], also associated with *IFIH1* in humans. Contrary to the results of the motivating study in mice, we see no association of soluble mediators with SNPs in exon 13 of *IFIH1*. When we expanded our analyses, however, to include 135 SNPs across *IFIH1* we found two promising effects associated with IL-6 and IP-10. Although previous investigations have shown *IFIH1*, IL-6, and IP-10 to be associated with SLE [4, 19, 20], none have shown a direct connection between human *IFIH1* and these two soluble mediators. SNPs significantly associated with IL-6 (rs76162067) and IP-10 (rs79711023) are located within the region of exons 1–3 that encodes the CARD1/CARD2 domain of the MDA5 protein [4]. The CARD1/CARD2 domain of *IFIH1*-encoded MDA5 has been shown to signal through the adaptor protein, interferon-β promoter stimulator (IPS-1) [21], necessary for MDA-5 mediated secretion of IL-6 (via NFκB) and IP-10 (via NFκB and IRF3) [22].

We have recently demonstrated that IL-6 [23] and IP-10 [24] are altered early in pre-clinical SLE pathogenesis, prior to the onset of clinical disease, and additional alterations occur again prior to imminent clinical disease flare in patients with classified SLE [14]. In addition to the two genetic variants associated with IL-6 and IP-10 in the current study, SNP rs13023380 has recently been associated with the CARD1/CARD2 encoding region of *IFIH1* in SLE patients [4] and lies closest to the IL-6 and IP-10 associated SNPs in our *IFIH1* LD map. These findings suggest that the IPS-1 binding region MDA5 is significantly associated with SLE and may contribute to altered IL-6 and IP-10 levels. We observed that those subjects with the minor allele for rs76162067 tended to have higher levels of IL-6, while those with the minor allele for rs79711023 tended to have lower levels of IP-10, including both SLE patients and FDRs of SLE patients, who are at increased risk of transitioning to classified disease [6, 25]. These results suggest that genetic variants within *IFIH1* may directly contribute to alterations in the levels of inflammatory mediators associated with SLE pathogenesis and will be the subject of future study.

When we stratified the soluble mediator data by disease status, as mentioned previously, unaffected FDRs of SLE patients not included in the current study had the highest plasma levels of IL-6 and IP-10, significantly higher than both cases and unrelated, unaffected controls, independent of medication usage. These data suggest that FDRs have some level of immune dysregulation that may be partially genetically driven by *IFIH1*, as has been previously shown for SLE patients [4, 5]. We have recently shown that these unaffected FDRs also displayed the highest plasma levels of the regulatory mediator IL-10 compared to FDRs who transitioned to classified SLE and unrelated, unaffected healthy controls, independent of medication usage [6]. This suggests that although FDRs of SLE patients exhibit pro-inflammatory immune dysregulation compared to unrelated controls, they have heightened regulatory activity that may allow them to remain unaffected, as these FDRs have been followed for an average of 6.7 years without transition to classified SLE [6].

The lack of direct replication between mouse and human genetic associations can occur for a number of reasons. First, differences in linkage disequilibrium (LD) may explain the absence of a significant association within exon 13 as we are not directly observing all possible variation in human *IFIH1*. LD is roughly a measure of how often alleles are inherited together. If, for example, certain alleles are commonly inherited together in mice at a different frequency than they are in humans, we would expect to see differences between species. Although conservation of haplotype structure across mammals has previously been reported on a genome-wide scale, LD block size and structure at the gene/exon level is indeed species-dependent [26]. For instance, the significant exon 13 variants previously reported in *Mda5* may be in LD with a causal variant in the mouse model but not so with the human model. A limitation of the current study is that only 135 of the 1824 *IFIH1* variants identified in dbSNP [27] were available for analysis. It is possible that some additional, potentially significant SNPs around exon 13 were not sequenced or that a discrepancy in LD structure failed to capture the association signal. Previously identified SNPs shown to be significantly associated with SLE [4, 5] were found to lie downstream of rs76162067 and rs79711023 on our LD map. This is not surprising given that those SNPs were pinpointed using case-control analyses, rather than analysis based on immune mediator phenotype in the current study. That being said, it is quite exciting that the two SNPs we identified in the current study are located in the region of *IFIH1* known to encode the CARD1/CARD2 domain that drives MDA5-mediated IL-6 and IP-10 production, as well as lying in proximity of a previously identified SNP associated with SLE that also lies within the CARD1/CARD2 domain encoding region of *IFIH1* [4].

Despite differences between our findings and those of Funabiki et al., associations of genetic variants with a common physiological function have been noted in mouse and human models containing modest phenotypic differences between the two species [28–30]. As such, the results of both our investigation and the Funabiki study support the role of *IFIH1* in autoimmune disease through its potential to influence dysregulation of inflammatory pathways. Future studies will build upon our exciting findings that SLE-associated mediators IL-6 and IP-10 are associated with genetic variants of *IFIH1* that directly impact MDA5-mediated secretion of these soluble mediators in SLE patients and their blood relatives. Of particular interest will be the potential effects of these *IFIH1* variants on the ability of MDA5 to respond to dsRNA and changes in signaling pathways that lead to IL-6 and IP-10 secretion that contribute to altered risk of either developing SLE or having increased morbidity due to a predisposition for altered disease activity in patients with classified SLE.

## Supporting information

S1 Fig Altered IL-6 and IP-10 levels in SLE patients and lupus relatives irrespective of medication. Comparison of IL-6 and IP-10 levels between each study group assessed by medication usage.
(PPTX)

S2 Fig Altered innate mediators and SLE-associated autoantibodies in SLE patients and lupus relatives. Comparison of mediators and autoantibodies between each study group.
(PPTX)

S1 Table R^2^ measure of LD. R^2^ values generated from LDmatrix module in the LDlink program referenced from all European populations in 1000 Genomes.
(XLSX)

S2 Table Medication Use in Cases, FDRs, and Controls. Counts and percentages of medication use within each study group.
(XLSX)
