# Supplementary material for: Association of IFIH1 and pro-inflammatory mediators: Potential new clues in SLE-associated pathogenesis
Source: PLoS One. 2017 Feb 24;12(2):e0171193. doi: 10.1371/journal.pone.0171193 (PMC5325200; doi:10.1371/journal.pone.0171193)
Supplement: S1 Fig — Comparison of IL-6 and IP-10 levels between each study group assessed by medication usage. (PPTX) [file pone.0171193.s001.pptx]

## Slide 1
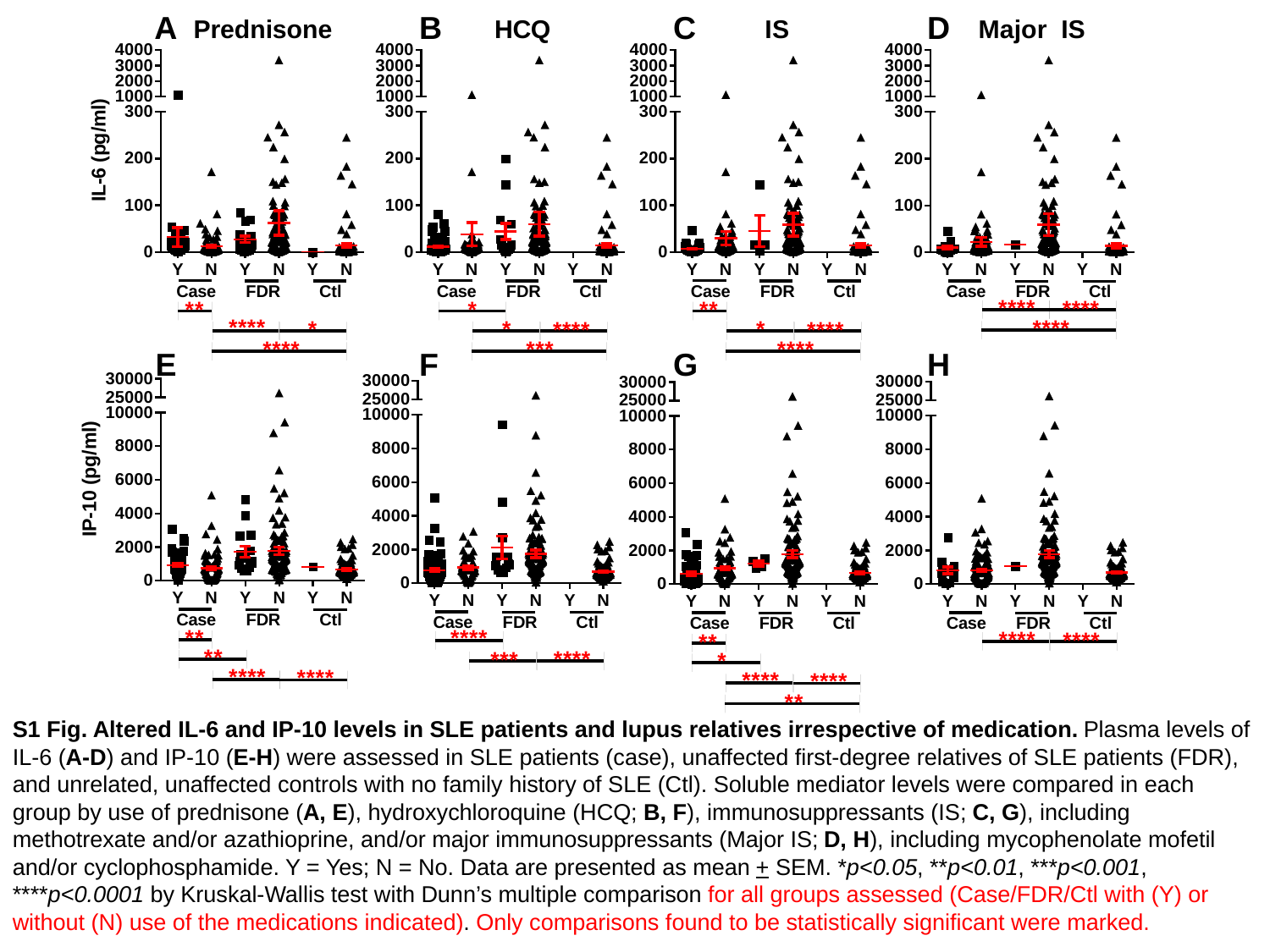

A
B
C
D
E
F
G
H
S1 Fig. Altered IL-6 and IP-10 levels in SLE patients and lupus relatives irrespective of medication. Plasma levels of IL-6 (A-D) and IP-10 (E-H) were assessed in SLE patients (case), unaffected first-degree relatives of SLE patients (FDR), and unrelated, unaffected controls with no family history of SLE (Ctl). Soluble mediator levels were compared in each group by use of prednisone (A, E), hydroxychloroquine (HCQ; B, F), immunosuppressants (IS; C, G), including methotrexate and/or azathioprine, and/or major immunosuppressants (Major IS; D, H), including mycophenolate mofetil and/or cyclophosphamide. Y = Yes; N = No. Data are presented as mean + SEM. *p<0.05, **p<0.01, ***p<0.001, ****p<0.0001 by Kruskal-Wallis test with Dunn’s multiple comparison for all groups assessed (Case/FDR/Ctl with (Y) or without (N) use of the medications indicated). Only comparisons found to be statistically significant were marked.
