# Supplementary material for: Association of IFIH1 and pro-inflammatory mediators: Potential new clues in SLE-associated pathogenesis
Source: PLoS One. 2017 Feb 24;12(2):e0171193. doi: 10.1371/journal.pone.0171193 (PMC5325200; doi:10.1371/journal.pone.0171193)
Supplement: S2 Fig — Comparison of mediators and autoantibodies between each study group. (PPTX) [file pone.0171193.s002.pptx]

## Slide 1
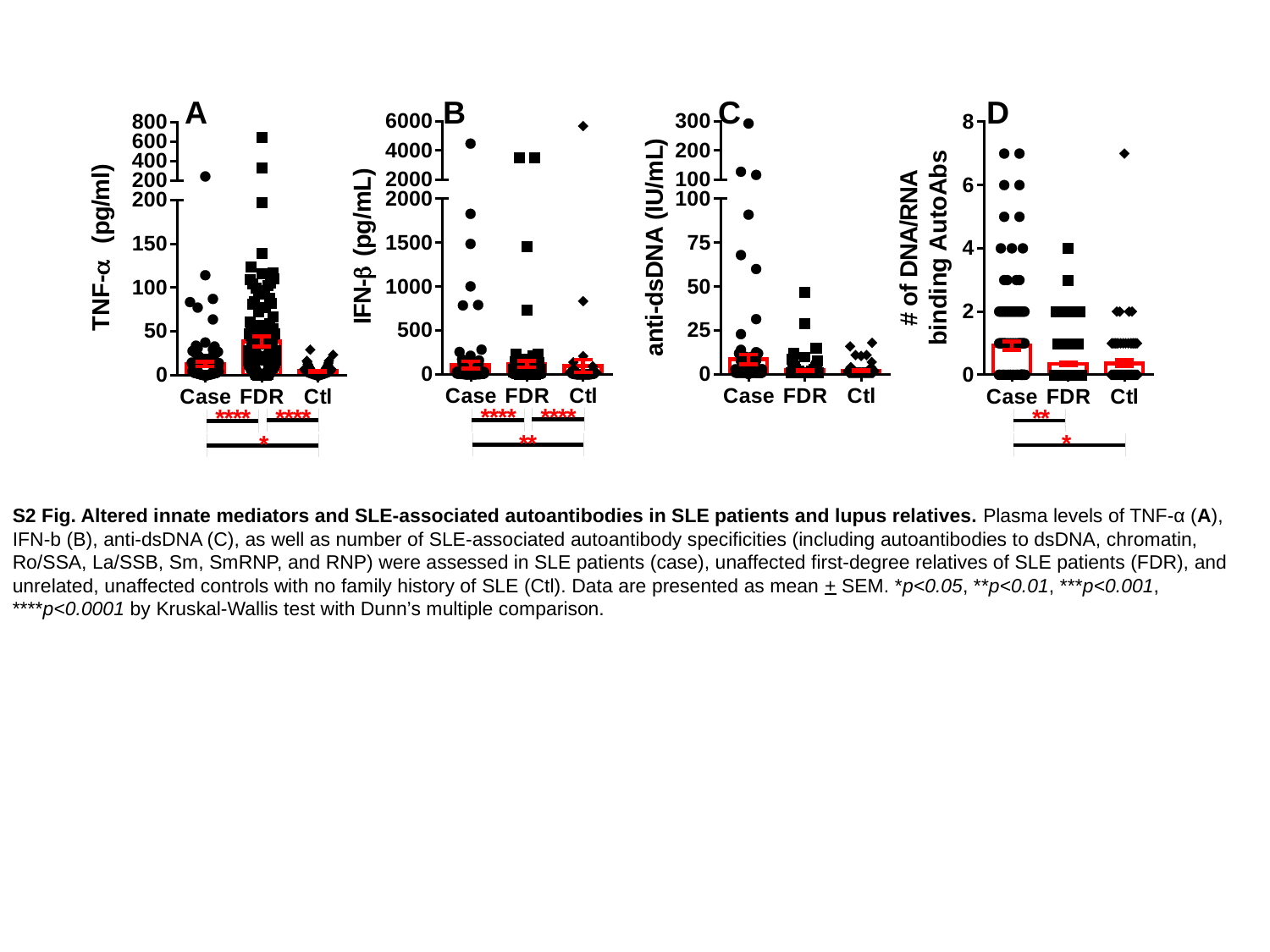

A
B
C
D
S2 Fig. Altered innate mediators and SLE-associated autoantibodies in SLE patients and lupus relatives. Plasma levels of TNF-α (A), IFN-b (B), anti-dsDNA (C), as well as number of SLE-associated autoantibody specificities (including autoantibodies to dsDNA, chromatin, Ro/SSA, La/SSB, Sm, SmRNP, and RNP) were assessed in SLE patients (case), unaffected first-degree relatives of SLE patients (FDR), and unrelated, unaffected controls with no family history of SLE (Ctl). Data are presented as mean + SEM. *p<0.05, **p<0.01, ***p<0.001, ****p<0.0001 by Kruskal-Wallis test with Dunn’s multiple comparison.
